# Quality of Life and Social Support of People on Peritoneal Dialysis: Mixed Methods Research

**DOI:** 10.3390/ijerph17124240

**Published:** 2020-06-14

**Authors:** Miquel Sitjar-Suñer, Rosa Suñer-Soler, Afra Masià-Plana, Emilia Chirveches-Pérez, Carme Bertran-Noguer, Concepció Fuentes-Pumarola

**Affiliations:** 1 University Hospital Dr. Josep Trueta, Nephrology Service, 17007 Girona, Spain; miquel.sitjar@hotmail.es; 2 UVic-UCC, Barcelona, 08500 Vic, Spain; 3 Department of Nursing, University of Girona, 17003 Girona, Spain; afra.masia@udg.edu (A.M.-P.); carme.bertran@udg.edu (C.B.-N.); concepcio.fuentes@udg.edu (C.F.-P.); 4 Health and Health Care Research Group, University of Girona, 17003 Girona, Spain; 5 Department of Nursing, UVic-UCC, 08500 Vic, Spain; emilia.chirveches@uvic.cat; 6 Research Group on Methodology, Methods, Models of Health and Social Outcome, UVic-UCC, 08500 Vic, Spain

**Keywords:** chronic kidney disease, peritoneal dialysis, social support, quality of life, renal nursing, research in practice, mixed methods

## Abstract

Although some study has been made into quality of life in patients with peritoneal dialysis, little is known about how this relates to social support. The aim of this paper was to study health-related quality of life, perceived social support and the experiences of people receiving peritoneal dialysis. A cross-sectional study was conducted using quantitative and qualitative methodologies, between June 2015 and March 2017. Fifty-five patients receiving peritoneal dialysis were studied. The most affected quality of life dimensions were the effects of the disease, the burden of the disease, occupational status, sleep and satisfaction. The physical component of the quality of life questionnaire was negatively associated with the number of hospital admissions over the previous year (*p* = 0.027) and positively associated with social support (*p* = 0.002). With regard to the mental component, age (*p* = 0.010) and social support (*p* = 0.041) were associated with a better quality of life. Peritoneal dialysis, while not a panacea, is experienced as being less aggressive than hemodialysis, allowing greater autonomy and improved perceived health. Greater symptomology corresponded to worse quality of life and to perceiving the disease as a burden. Patients had to adapt to the new situation despite their expectations. Social support was observed to be a key factor in perceived quality of life.

## 1. Introduction

Present day increases in life expectancy, considered a success of public health policy and socioeconomic development, bring with them the proliferation of chronic diseases that increase with age [1]. Chronic diseases are those which most lead to morbidity and death (responsible for almost 70% of deaths worldwide). Furthermore, they usually last a long time, have an uncertain prognosis and affect people of all ages [2].

Knowledge of the quality of life of patients with chronic diseases has become an indicator of the evolution of the state of health and enables integral treatment. Furthermore, this indicator allows us to understand the impact of the disease and its treatment, to know more about patients, how they evolve and how they adapt to the organic alteration. It also permits the prediction of future adverse consequences, evaluates treatment efficiency, helps in decision-making, and facilitates rehabilitation [3,4,5].

Among the modulating factors of the impact of chronic disease, social support stands out as having an effect which is directly proportional to an individual’s health [6,7,8] and lower rates of morbidity and mortality [9]; it acts as an absorber of the effects of psychosocial and physical stress and contributes to the promotion of healthy behavior, as well as the control and adaptation of chronic diseases [10,11,12,13,14,15]. It is also influential in the process of adaptation to the disease and health recovery [16]. Although quality of life in patients with peritoneal dialysis has been more extensively studied, there has been little study into how this relates to social support. In this respect, the present study investigated the quality of life, social support and the experiences of a group of people with chronic kidney disease who receive peritoneal dialysis therapy.

Chronic kidney disease (CKD) affects around 11% of the world’s adult population, with an increase in the incidence in recent years [17]. The age-standardized global prevalence of CKD in adults aged 20 and older was 10.4% in men and 11.8% in women [18]. This disease, which is considered a public health problem [19], results in sufferers having to use renal replacement therapy to substitute the renal function. There are currently two dialytic techniques available: hemodialysis and peritoneal dialysis [20]. The vast majority of studies have been undertaken in people who use hemodialysis, despite different authors reporting significant benefits in the quality of life for patients receiving peritoneal dialysis rather than hemodialysis [21,22,23,24,25]. The positive effects of home-based peritoneal dialysis include the preservation of residual renal function, permitting a less restrictive diet and conserving vascular accesses [26,27,28,29], and greater compatibility with leisure and daily life activities [23]. Specifically, the management and timetable flexibility for performing this therapy has been identified as a factor that is valued by people receiving peritoneal dialysis and which contributes to their satisfaction with the treatment and their adherence to it [30]. Other investigators who have studied the experiences of these people have observed similar results from qualitative studies [31], as well as a sensation of self-sufficiency and greater freedom with this therapy [31,32].

A further benefit is the significantly lower cost of peritoneal dialysis, which in our region is on average EUR 14,000 per year less than for hemodialysis, demonstrating that investing in peritoneal dialysis is beneficial for the national health system [33,34,35].

The quality of life of people with chronic renal insufficiency receiving peritoneal dialysis treatment is affected by both the chronic disease and the treatment, especially the dimensions referring to problems and symptoms caused by kidney disease, the burden of the disease itself and the ability to continue working [36,37,38,39]. More time on dialysis treatment was also associated with poorer perceived health [40,41]. In contrast, social support is valued positively in these people, especially family support and resources [42,43].

The main purpose of this research was to study the health-related quality of life and perceived social support of people receiving peritoneal dialysis with the hypotheses that the perception of quality of life related to the health of people with chronic renal insufficiency and that peritoneal dialysis is related to the symptoms of the disease and social support. The second aim was to describe the experience of people who receive peritoneal dialysis with the hypothesis that peritoneal dialysis treatment is experienced as a less aggressive treatment that allows greater autonomy.

## 2. Methods

### 2.1. Design

A cross-sectional multicenter hospital-based study, using quantitative and qualitative methods, was carried out between June 2015 and March 2017 in order to achieve an integrated evaluation corresponding to the reality of the experiences of people on peritoneal dialysis [44].

### 2.2. Setting and Participants

The sample consisted of all those people with chronic renal insufficiency who were at least 18 years old, had received three months or more of renal replacement therapy in the Health Girona Region (Catalonia, Spain) through peritoneal dialysis at the time of data gathering, and who understood Spanish, and spoke either Catalan or Spanish.

The study variables were sex, age, perception, economic status, level of studies, lifestyles, months of dialysis treatment, years of disease progression, number of hospital admissions in the previous year, biological problems and symptoms, unpleasant effects caused by the renal disease, burden due to kidney disease, occupational status, cognitive function, quality of social interaction, sexual function, sleep, social support, dialysis personnel, patient satisfaction, physical functioning, physical role, presence of pain, general current and future perception of health, mental health, emotional role, social function, energy, and fatigue.

### 2.3. Data Collection

The study sample was obtained from those included in the peritoneal dialysis program. New cases were added during the study period after three months of treatment. Before their inclusion, potential participants were informed about the study and that participation was voluntary during a scheduled visit or by telephone. Once a person had agreed to participate, the date and time of the collection of data were decided, when possible coinciding with a planned hospital visit. Clinical data were obtained from the patient’s clinical history through each center’s electronic clinical data bases. In addition, the participants were invited to participate in a focus group. Invitations to participate were made by telephone to all the people who were actively receiving treatment and fulfilled the study’s inclusion criteria. The final number of participants in the focus group was 10 (5 men and 5 women, falling within the ideal range of 4 to 12 people established by [45] Holloway and Galvin (2016) for such interviews. As elements of the study, the participants responded about their experience with dialysis therapy, perceived quality of life, social support, and satisfaction with care.

### 2.4. Instruments

The Kidney Disease Quality of Life instrument version 1.3 published by Hays et al. [46] and validated by Alvarez-Ude et al. [47] in Spain was used to study the quality of life of people with renal insufficiency on dialysis.

In order to find out the level of social support, the MOS social support survey published by Sherbourne et al. [48], and validated by Revilla et al. [49] for Spain, was used.

The qualitative study was conducted through a focus group, as it enabled a deeper examination of the subject of interest of the research with regard to the elements of social support and perceived quality of life through the opinions, beliefs and experiences of the participants, so obtaining information that would be less accessible without the connection of the group.

#### Validity and Reliability of Instruments

The Spanish version of the Kidney Disease Quality of Life instrument (KDQOL-SF^TM^) [47] showed high internal consistency with a Cronbach alpha for each dimension of between 0.67 and 0.87 and of 0.93 for the questionnaire as a whole. No differences were found between the mean values of the first and second administration of the KDQOL-SF^TM^, and the intraclass test–retest correlation coefficients oscillated between 0.62 and 0.77. The validation of the Spanish version of the social support MOS questionnaire [49] confirmed its reliability. Variant analysis revealed the existence of three factors that explained the 68.72% global variance. All three showed values close to 1 (maximum consistence).

The focus group script was made after a literature review to identify the elements that could be studied.

### 2.5. Ethical Considerations

This project respects the Helsinki Declaration of the World Medical Association on the ethical principles for medical investigations into humans. Informed consent was obtained from all individual participants included in the study. The confidentiality of the participants has been maintained at all times. The study was approved by the Clinical Research Ethics Committee (code IRC-Dialysis) and complied with the Strengthening the Reporting of Observational Studies in Epidemiology (STROBE) checklist.

### 2.6. Data Analysis

#### 2.6.1. Quantitative Data

The Statistical Package for the Social Sciences (SPSS) version 25.0 (IBM, Madrid, Spain) was used for data analysis. Quantitative variables are expressed with the mean and standard deviation or the median and the interquartile range, and categorical variables with the absolute frequency and percentage. The Chi-squared test was used to study the associations between categorical variables and the Mann–Whitney-U non-parametric test was used to compare two independent groups. Correlations between the dimensions of quality of life and social support were made using Spearman’s Rho test. In order to study the factors associated with perceived quality of life both physically and mentally, two multiple linear regression models were performed. In these models, social support was analyzed through the total score of the questionnaire, rather than with its subscales, in order to avoid collinearity. Significance is taken as *p* < 0.05.

#### 2.6.2. Qualitative Data

For the analysis of data during the qualitative phase, the transcribed information was ordered literally following three phases—reduction, categorization and encoding—maintaining the representation of the content through the classification by themes in accordance with the objectives of the study [50]. From the responses of the participants, different themes of analysis were constructed following the previously described steps. With the aim of guaranteeing the interrater reliability of the themes, the process of categorization of the data was performed independently by the three investigators. The analysis was compared and debated in meetings in which each investigator acted as a third judge. The criterion established to consider a theme as valid was that at least two of the three researchers agreed.

## 3. Results

### 3.1. Results of the Quantitative Phase of the Study

All 55 patients who were receiving renal replacement therapy through peritoneal dialysis at the time responded to the questionnaires. All the participants at the time of data gathering had at least one other associated chronic disease apart from the renal condition: 34.5% had high blood pressure, 18.7% had dyslipidemia and 15.8% had diabetes. The rest of the demographic characteristics of the participants are given in Table 1.

#### 3.1.1. Kidney Disease Quality of Life 

When comparing the different dimensions of the KDQOL-SF^TM^ specific questionnaire (Table 2), it was observed that the dimensions of the effects of kidney disease, burden of kidney disease, work status, sleep and patient satisfaction were the dimensions that obtained the lowest scores. On the other hand, the dimensions of present-day problems and symptoms, cognitive function, quality of social interaction, sexual function, social support, dialysis staff encouragement, and patient satisfaction with regard to the staff were the dimensions that obtained the best scores. When comparing the different dimensions by sex, it was observed that men reported having more problems in sexual function than women (*p* = 0.023), and these showed more satisfaction with their relationship with the staff (*p* = 0.042). With regard to the eight dimensions of the SF-36 questionnaire, emotional role and pain obtained the highest scores. On the other hand, the lowest scores were obtained for the dimension of general health and vitality, without significant differences by gender. 

#### 3.1.2. Social Support

On correlating the size of the social network and the different dimensions of the MOS questionnaire, it was observed that people with a large social network show a higher score in the dimension of positive social interaction (Spearman’s Rho = 0.395; *p* <0.001). However, there was no correlation between the size of the social network and the dimensions of emotional/informational support (Spearman’s Rho = 0.192; *p* = 0.160), instrumental support/material support (Spearman’s Rho = 0.166; *p* = 0.225) and emotional support (Spearman’s Rho = 0.147; *p* = 0.286).

In comparing the dimension of physical limitations of the KDQOL-SF^TM^ questionnaire with the dimensions of social support of the MOS (see Table S1), it is observed that people who did not have to reduce the time devoted to their activities had slightly higher scores in the different dimensions of social support, and those who did not do less than they wished to obtained slightly higher scores in the dimensions of emotional support, instrumental support, positive social interaction and emotional support, with significant differences depending on the instrumental support dimension (*p* = 0.001). People who did not have to stop performing some tasks in their work or daily activities obtained slightly higher scores in the different social support dimensions. People who did not have to stop doing daily activities had higher scores in instrumental support (*p* < 0.001). People who did not do less work than they wished to obtained slightly higher scores in the dimensions of emotional support, instrumental support, positive social interaction and emotional support.

When the emotional influences of the KDQOL-SF^TM^ instrument were related to the social support dimensions of the MOS questionnaire (Table S2), it was seen that people who did not reduce the time spent on their activities due to emotional problems presented slightly higher scores in the different dimensions of social support, with significant differences depending on the instrumental support dimension (*p* = 0.005). People who did not do less than they would have wished for an emotional problem achieved slightly higher scores in emotional support, instrumental support, positive social interaction and emotional support. People who did not do fewer tasks than they would have liked to for some emotional problems obtained slightly higher scores in the different dimensions of social support.

By correlating the dimensions of quality of life, (KDQOL-SF^TM^) and social support, (MOS) (Table 3 and Table S3), it was observed that the physical component correlated above all with the symptoms of the disease (Spearman’s Rho of 0.437; *p* = 0.001), with the higher the score in this dimension (in other words, fewer symptoms), the higher the score in the physical component of the SF-36. With regard to the mental component, it mostly correlated with the burden of the disease, with the higher the score in this dimension (less burden of kidney disease), the higher the score in the mental component of the SF-36 (Spearman’s Rho of 0.649; *p* <0.001).

Finally, in the linear regression model to study the factors associated with perceived quality of life with regard to physical factors, an inverse association is found with the number of hospital admissions in the previous year (*p* = 0.027) and social support is directly and associated with perceived health (*p* = 0.002). Age is also associated with perceived health, with a tendency towards significance (Table 3). The factors associated with quality of life with regard to mental factors are age (*p* = 0.010) and social support (*p* = 0.041) (Table 4).

### 3.2. Results of the Qualitative Phase of the Study

The discussion group, formed of ten patients with renal replacement therapy, allowed to identify seven themes which emerged through the patients’ related reflections.

#### 3.2.1. Theme 1: Perception of the Dialysis Treatment

Several members of the group who were familiar with hemodialysis either through their own experience or through that of a close relative considered peritoneal dialysis to be a less aggressive dialytic technique and perceived it as giving greater autonomy and a greater perception of good health than hemodialysis.

*“I noted a huge change in comparison with hemodialysis ... because I find hemodialysis very hard, I felt terrible. I used to do it in the morning and in the afternoon, I was terribly tired”*.

(Participant 3)

#### 3.2.2. Theme 2: Physical Health and Repercussions on the Perceived Quality of Health

It was seen that the kidney disease itself had an effect on those who suffered from it, and that these effects could be accentuated by the treatment. Several reporters said they had less energy, less strength and more fatigue when required to make small efforts.

*“...physically, at least in my case, it affects us a lot, in all ways. You get up in the morning and the sensation that you have of being full of energy little by little disappears, occasionally you have this sensation a little longer, but normally if you don’t take a nap at lunchtime, by the afternoon you feel a wreck”.*

(Participant 1)

They mentioned that they had to compensate their lack of energy by doing other activities or making compensatory breaks during their activity.

*“... I like to go to the woods, to run in the mountains, but now I can’t go where I used to. I can’t even climb a little. When I have gone six meters upwards, I think I don’t know if I will be able to come down and you have to sit down and say to yourself “oh well, take it easy”.*

(Participant 7)

#### 3.2.3. Theme 3: Psychic Health and Repercussions on the Perceived Quality of Health

It was seen that ignorance and misunderstanding among people around the patient sometimes caused negative feelings in the patient; these repercussions were present in the mental conflicts they experienced.

*“One day I was with my mother-in-law and we met a woman ... she looked at me and said, “You are so chubby and good-looking that I don’t even need to ask you how you are”. I was very upset because I am definitely fat. The bad thing is that I am overweight and I would like to be thinner, but I can’t. And they think that because I am like this that there is nothing wrong with me”*.

(Participant 6)

#### 3.2.4. Theme 4: Social Support

Different participants considered that the disease and the treatment itself had social repercussions on the quality of life, above all due to the limitations of renal replacement therapy in daily life.

*“I feel a bit restricted because I can’t go on excursions freely. I can’t say, for example, today I will stay over somewhere...I have to go home to sleep. I can’t go away for a weekend, well I could with the handheld bags but it’s such a bother that in the end you say that it’s best not to complicate your life”*.

(Participant 3)

The patients commented that they had had to make adaptations in their lives because the complexity of the treatment affected the work that they had before becoming ill.

*“Me no, since I have been doing it the truth is that I haven’t gone anywhere. I haven’t spent a single night away. We used to go away for 8 days..., this has finished” (Participant 6). “I have had to stop working, I used to do morning, afternoon and night shifts”*.

(Participant 4)

As the participants mentioned, it was very difficult to share their experiences, limitations and the discomfort caused by the disease and the treatment with other people.

*“...I normally don’t try to explain to friends, I don’t try to explain what I have because they don’t understand. So no, I don’t explain, and I pretend that everything is fine, that everything continues as normal and I leave it at that”*.

(Participant 1)

#### 3.2.5. Theme 5: Rest and Sleep

The participants reported that due to the treatment being normally performed at night to avoid intrusion in daily activities, they often could not rest well, whether due to problems associated with the treatment itself or by pain caused in the infusion and drainage of dialytic solutions.

*“... yes, and at night also, when I sleep during the day straight through, fantastic, but there are many nights when perhaps the drainage is difficult and, of course, I am in pain and I can’t sleep. For this reason, when you disconnect the machine, you sleep deeply for two hours...sometimes I even sleep at work. Literally I fall asleep. And I have to get up because otherwise they would find me asleep”*.

(Participant 3)

#### 3.2.6. Theme 6: Adaptation to the Disease and Treatment

Several members of the group explained that the complexity of the technique resulted in them having to modify the family residence in order to fit in all the necessary material to be able to perform the therapy.

*“I removed the cupboard from the dining room, I put up two shelves and when the deliveryman came there was space for him to put the equipment there”*.

(Participant 2)

Being ill challenged patients to reinvent themselves to meet the demands of their immediate surroundings and find a balance between their expectations and the situation that they were experiencing.

*“I have to spread things out, we could say, it’s not like before when I would do 40 things in the morning and then head off for work. No, now it’s different, the day that I go shopping, I go shopping, the day that I do the washing, I do the washing. But if I try and do several things I get very tired”*.

(Participant 3)

However, once the treatment was incorporated in the daily routine and with the help of new technologies, most participants indicated that the therapy was not a very bad experience.

*“At first, no. It is difficult to get used to and little by little you learn the system...you also discover things, they are new strategies, I spend my time visiting patients and the elderly and I have discovered a lot by telephone ... sometimes you are not in a good enough condition to go and make three visits one afternoon and instead you decide to do it by telephone. It is not the same as a personal visit, but it is very helpful nonetheless”*.

(Participant 5)

However, during long periods of treatment it could be increasingly hard and frustrating, causing new imbalances between planned expectations and the reality of coexistence with the treatment.

*“I think that as the process has advanced it has become more tiring. Now I have been doing it for three years. At the beginning I didn’t find it so tiring, I took to it quite well. Well, I don’t know, perhaps at first I found it a little worrying, but then once you get used to it, it really isn’t. I find that with time it becomes a little tiring. A bit hard... I can’t lead a normal life, if I evaluate the life that I led before all of this, when we would do 40 things and then, all of a sudden, I had to give up everything”*.

(Participant 4)

#### 3.2.7. Theme 7: Attention Received from the Health Professionals

In general, the participants commented that their experience with the health professionals who took care of them throughout the treatment process was good, especially in the learning process, which facilitated the communication. Participants also experienced the healthcare they received in the individual periodic controls of the treatment and perceived that the professionals at these scheduled appointments were exclusively focused on them.

*“My experience is fantastic”*.

(Participant 7)

*“... the support of professionals is very important (...) during the learning period”*.

(Participant 5)

*“I am very happy with the way I have been treated ... I feel that when I arrive that I am the only one there, that’s the sensation I have”*.

(Participant 4)

## 4. Discussion

People referring to more symptoms reported lower perceived physical quality of life. Likewise, the greater the perception of the burden of chronic renal insufficiency, the lower the perceived mental quality of life. The higher percentage of men than women in this research was in line with other investigations [51,52]. The mean age of the participants of 61.4 years was 7 years higher than the mean age of people on peritoneal dialysis according to Remón et al. [53]. However, recently published studies have reported mean ages that are similar to the present investigation [52,54].

The average score for the perception of the economic situation was of five points; this perception could be explained by the fact that 83.6% of participants did not work. In the focus group, some patients explained that they had to stop working because they could not combine work and treatment. In the study by Law et al. [55], these data were corroborated, since only 8.6% of patients receiving renal replacement therapy through peritoneal dialysis maintained full-time work and 4.4% worked part-time. According to Julian et al. [27], only 33.3% of patients of working age receiving renal replacement therapy were in active employment.

It was found that more than three quarters of the patients had been suffering from a kidney problem for at least five years which had evolved until requiring renal replacement therapy. Some authors, such as Heras et al. [56], found controversies in establishing the time of evolution of the disease.

In our investigation, the participants were hospitalized on average at least once during the previous year and this variable was significantly associated with perceived health with regard to physical factors. Perl et al. [57] observed that patients receiving home peritoneal dialysis treatment 30 days after hospital discharge were more likely to be readmitted compared to those who received hemodialysis. Specifically, the focus group patients perceived that peritoneal dialysis was a less aggressive dialysis technique that allowed for greater autonomy and better perception of health than hemodialysis. The results obtained can be compared to those of the study of Almutary et al. [58], who observed that patients receiving peritoneal dialysis as renal replacement therapy rather than hemodialysis were more satisfied with the treatment, perceived having greater autonomy, maintained better cognitive functions, and had the perception that the treatment was shorter.

An average of 75.64 points was obtained for the first dimension of the KDQOL-SF^TM^ scale, referring to the problems and symptoms presented by the patients due to kidney deterioration. These results are similar to those found by Lim et al. [59] and Gonçalves et al. [60]. The average score for the second dimension of the KDQOL-SF^TM^ scale, referring to the effects of kidney disease, was 65.39 points, which is similar to that found by Lim et al. [59], although their scores were slightly higher. Referring to these results, focus group participants stated they had less fatigue and increased energy in making small efforts. On the other hand, the lower scores regarding the effects of renal disease in daily life reported in Griva et al. [61], Tannor et al. [62], and Zouari et al. [63] show that their patients were more discomforted than ours. In the third dimension of the ESRD instrument, referring to the burden of kidney disease, the average score of 41.93 points confirmed that the disease had an impact on people receiving renal replacement therapy. This impact was recently seen in the systematic review and meta-analysis of Ghiasi et al. [64]. Our results are between 1 and 25 points lower than those obtained by Gonçalves et al. [61], Czyzewski et al. [65], Tannor et al. [62] and Zoauri et al. [63], whose subjects reported less of a burden associated with receiving renal replacement therapy than our patients. On the other hand, Lim et al. [59] and Park et al. [66] found that the burden of renal disease was greater than the results obtained in the present investigation. In the fourth dimension of the KDQOL-SF^TM^ scale referring to work status, the average score of 37.27 points highlighted the fact that a significant number of patients did not work due to the illness and the renal replacement therapy. Similar scores were observed in Tannor et al. [62], while slightly lower scores were found in Oliveira et al. [67]. On the other hand, in the studies of Gonçalves et al. [61] and Czyzewski et al. [65], higher scores were recorded for people who could work but did not do so as a result of the renal replacement therapy. With regard to the second section of the KDQOL-SF^TM^ instrument evaluating the eight dimensions of the perceived quality of life of the SF-36 instrument, the average score of the physical component is approximately six points lower than the general population and four points lower for the mental component [68,69].

The MOS instrument was used to study the social support of people receiving renal replacement therapy. The overall social support score (78.27 points) revealed that those surveyed perceived receiving optimal social support. The present study observed that the perceived social support of the participants is significantly associated, both physically and mentally, with perceived health. In this respect, we have not been able to find similar studies to compare the relationship between perceived health in both physical and mental terms with social support, but other authors have observed that social support is independently associated with self-management behaviors in people with chronic kidney disease [70].

On the other hand, our focus group concluded that they did not feel understood by the people around them, except direct relatives, and they avoided talking about the disease and hid their suffering. In this respect, Li et al. [71] and Wang et al. [72] reported a poor perception of social support. These results could be conditioned by the excessive burden on informal caregivers as described in Hoang et al. [73].

### 4.1. Limitations

The main limitation of this study is the methodology used; the cross-sectional design allowed the study of social support and perceived quality of life of people with peritoneal dialysis and their relationships, but did not allow for causal relationships to be established between the variables studied. In this respect, longitudinal design studies permit better visibility of the evolution of the perception of health in these people with chronic renal failure. Another limitation is the difficulty in integrating the results of the different methodologies used, although the results obtained do enrich the study overall, providing a better understanding of people living in situations of chronicity with complex treatments. The size of the sample is also a possible limitation, despite the participation of 100% of the population receiving renal replacement therapy through peritoneal dialysis in the region under study.

### 4.2. Practical Implications

The results indicate that the symptoms and effects derived from chronic renal insufficiency and peritoneal dialysis affect people’s perceived health, especially when people refer to the burden that suffering from this health problem represents for them. In this respect, it is highly advisable in the early phases of the disease and before starting a dialysis program to strengthen adaptation strategies to cope better with the treatment and the evolution of the disease. Furthermore, given the key role that social support also plays for people in this situation, it is recommendable to encourage the creation of patient-led groups from the health institutions themselves and/or from the community to share experiences and strategies, promote staying active in social relationships, aid understanding, and to assist in the healthy adaptation to each new stage of the disease.

## 5. Conclusions

The typical profile of the people included in our study was of a person diagnosed with chronic renal insufficiency five or more years earlier. The subjects were mainly men with a mean age of 61 years. The lowest health-related quality of life scores corresponded to the perception of general health, vitality and physical roles. The social support perceived by participants was optimal, and this support has been associated, both physically and mentally, with a better perceived quality of life.

People on peritoneal dialysis perceived the therapy as a less aggressive dialytic technique, allowing more autonomy and a greater perception of health, despite requiring a period of personal and environmental adaptation. Sometimes patients felt misunderstood by their close environment. On the other hand, they valued the treatment and information received from the health team positively.

## Figures and Tables

**Table 1:** Characteristics of the participants.

Variables	
Sex	
Men	38 (69.1)
Women	17 (30.9)
Mean age (SD)	61.4 (14.6)
Educational level	
No formal education	4 (7.3)
Primary studies	23 (41.8)
Secondary studies	19 (34.5)
Higher level studies	9 (16.4)
Perception of economic level (SD)	5.16 (1.8)
Lifestyle	
Does physical exercise	27 (49.1)
Does not do physical exercise	28 (50.9)
Months of dialysis treatment (SD)	22.9 (20.9)
Evolution of the disease	
Less than 5 years	13 (23.6)
5 years or more	42 (76.4)
Number of hospital admissions during last year (SD)	1.09 (1.4)

Continuous variables are described with the mean and the standard deviation in parentheses and categorical variables are described with the absolute frequency and their percentages by columns.

**Table 2:** Measures of central tendency and dispersion for the Kidney Disease Quality of Life (KDQOL-SF^TM^) instrument.

	Total Population N:55 Mean (SD) Median [IQR]	Men N:38 Mean (SD) Median [IQR]	Women N:17 Mean (SD) Median [IQR]	*p*
Problems and symptoms present	75.64 (13.82) 79.16 [68.75–87.5]	74.28 (15.25) 79.16 [59.37–84.37]	78.67 (9.61) 79.16 [75–87.5]	0.634
Effects of the kidney disease	65.39 (21.14) 65.62 [50–81.25]	64.06 (21.06) 65.62 [49.21–81.25]	68.38 (21.64) 68.75 [53.12–89.06]	0.500
Burden of the kidney disease	41.93 (22.96) 37.5 [25–56.25]	41.77 (21.11) 40.62 [25–56.25]	42.27 (27.37) 37.5 [21.87–68.75]	0.898
Work status	37.27 (34.98) 50 [0–50]	40.78 (34.59) 50 [0–50]	29.41 (35.61) 0 [0–50]	0.269
Cognitive function	77.57 (24.69) 86.66 [66.66–100]	72.80 (27.39) 80 [51.66–100]	88.23 (12.14) 93.33 [80–100]	0.102
Quality of social interaction	74.90 (21.69) 80 [66.66–93.33]	72.10 (23.60) 73.33 [58.33–88.33]	81.17 (15.49) 86.66 [66.66–96.66]	0.205
Sexual function	70.90 (34.94) 75 [50–100]	63.81 (37.07) 75 [34.37–100]	86.76 (23.58) 100 [75–100]	0.023
Sleep and satisfaction	65.68 (23.82) 70 [47.5–82.5]	64.34 (25.97) 68.75 [46.87–85.62]	68.67 (18.50) 70 [53.75–81.25]	0.799
Social support	76.05 (23.95) 83.33 [66.66–100]	74.55 (25.33) 74.99 [66.66–100]	79.40 (20.85) 83.33 [66.66–100]	0.436
Dialysis staff and encouragement	91.36 (11.99) 100 [75–100]	90.78 (12.22) 100 [75–100]	92.64 (11.74) 100 [75–100]	0.554
Satisfaction of patients with their relationship with the staff that attend them	85.75 (14.84) 83.33 [83.33–100]	83.33 (15) 83.33 [79.16–100]	91.17 (13.33) 100 [83.33–100]	0.042
SF-36 Dimensions				
Physical functioning	66.27 (25.38) 75 [50–85]	64.21 (25.82) 67.5 [48.75–86.25]	70.88 (24.50) 85 [55–87.5]	0.390
Physical role functioning	57.75 (40.78) 75 [25–100]	59.86 (40.91) 75 [18.75–100]	52.94 (41.34) 50 [12.5–100]	0.644
Bodily pain	68.13 (25.90) 70 [45–90]	68.48 (26.82) 73.75 [45–92.5]	67.35 (24.48) 67.5 [46.25–90]	0.776
General health perceptions	40.81 (18.85) 40 [25–50]	40.56 (18.22) 40 [30–50]	41.47 (20.74) 40 [25–57,5]	0.913
Mental health	66.76 (19.54) 72 [52–80]	67.47 (18.44) 72 [52–80]	65.17 (22.35) 68 [44–84]	0.675
Emotional role functioning	76.96 (37.88) 100 [66.66–100]	77.19 (38.83) 100 [58.33–100]	76.47 (36.82) 100 [50–100]	0.780
Social role functioning	63.86 (27.07) 62.5 [37.5–87.5]	62.5 (26.47) 62.5 [37.5–75]	66.91 (28.96) 75 [37.5–100]	0.592
Vitality	46.45 (21.40) 45 [30–60]	44.60 (21) 40 [30–60]	50.58 (22.35) 50 [32.5–67.5]	0.210

Mann-Whitney U test. The quantitative variables are described with the mean (standard deviation) and median [IQR].

**Table 3:** Regression model for perceived physical quality of life.

	Dependent Variable: Standardized Physical Component (SF-36 Questionnaire)				
	B	SE	CI 95%	β	*P*
Age	−0.127	0.068	−264–0.010	−0.227	0.069
Number of hospitalizations last year	−1.596	0.703	−3.008–−0.185	−0.278	0.027
Social support (MOS survey)	0.201	0.061	0.078–0.325	0.403	0.002
Corrected R^2^	0.248				

B: coefficient B; SE: standard error; CI 95%: confidence interval of 95%. β: standardized beta coefficient. Corrected R^2^: adjusted R-Squared (the adjusted coefficient of determination).

**Table 4:** Regression model for perceived mental quality of life.

	Dependent Variable: Standardized Mental Component (SF-36 Questionnaire)				
	B	SE	CI 95%	β	*p*
Age	0.278	0.104	0.069–0.487	0.329	0.010
Number of hospitalizations last year	1.370	1.071	−0.780–3.520	0.158	0.207
Social support (MOS survey)	0.196	0.094	0.008–0.384	0.260	0.041
Corrected R^2^	0.234				

B: coefficient B; SE: standard error; CI 95%: confidence interval of 95%. β: standardized beta coefficient. Corrected R^2^: adjusted R-Squared (the adjusted coefficient of determination).

## Supplementary Materials

The following are available online at https://www.mdpi.com/1660-4601/17/12/4240/s1, Table S1: Relationship between the physical limitations dimension of the KDQOL-SF^TM^ instrument and the social support dimensions of the MOS survey., Table S2: Relationship between the emotional limitation dimensions of the KDQOL-SF^TM^ instrument and the social support dimensions of the MOS survey., Table S3: Correlation between the physical and mental component of the SF-36, emotional support, instrumental support, affective support, cognitive function, sexual function, problems and symptoms, discomforting effects of the kidney disease, the burden of the kidney disease and social support.

## References

[1] Castelao A.M., Gorostidi M., Gorriz J., Olmo R.S., Bover J., Segura J. (2015). Reflexiones a propósito de dos documentos de consenso sobre enfermedad renal crónica. Nefrología.

[2] WHO (2018). Noncommunicable diseases and their risk factors. https://www.who.int/ncds/introduction/en/.

[3] Pérez T., Rodríguez A., Buset N., Rodríguez F., García M., Pérez P., Rodríguez J.C. (2011). Psiconefrología: Aspectos psicológicos en la poliquistosis renal autosómica dominante. Nefrología.

[4] Rubio A.R., Morales-Asencio J.M., Pons-Raventos M.E., Mansilla-Francisco J.J. (2015). Review of studies on health related quality of life in patients with advanced chronic kidney disease in Spain. Nefrología.

[5] Li Z., Zhang Y., Zhang L. (2017). Evolution and future trends of integrated health care: A scientometric analysis. Int. J. Integr. Care.

[6] Broadhead W.E., Kaplan B.H., James S.A., Wagner E.H., Schoenbach V.J., Grimson R., Heyden S., Tibblin G., Gehlbach S.H. (1983). The epidemiologic evidence for a relationship between social support and health. Am. J. Epidemiol..

[7] Chae Y.R., Lee S.H., Jo Y.M., Kang H.Y. (2019). Factors related to Family Support for Hemodialysis Patients: A Systematic Review and Meta-analysis. Korean J. Adult Nurs..

[8] Sousa H., Ribeiro O., Paúl C., Costa E., Miranda V., Ribeiro F., Figueiredo D. (2019). Social support and treatment adherence in patients with end-stage renal disease: A systematic review. Semin. Dial..

[9] Uchino B.N. (2006). Social Support and Health: A Review of Physiological Processes Potentially Underlying Links to Disease Outcomes. J. Behav. Med..

[10] Cassel J. (1976). The contribution of the social environment to host resistance: The Fourth Wade Hampton Frost Lecture. Am. J. Epidemiol..

[11] Cobb S. (1976). Social Support as a Moderator of Life Stress. Psychosom. Med..

[12] Ibrahim N., Teo S.S.L., Din N.C., Gafor A.H.A., Ismail R. (2015). The Role of Personality and Social Support in Health-Related Quality of Life in Chronic Kidney Disease Patients. PLoS ONE.

[13] Kaplan B.H., Cassel J.C., Gore S. (1977). Social Support and Health. Med. Care.

[14] Aristu A. (2000). Estrés y Emoción. Manejo e Implicaciones en Nuestra Salud de RS Lazarus.

[15] Schroevers M.J., Ranchor A.V., Sanderman R. (2003). The role of social support and self-esteem in the presence and course of depressive symptoms: A comparison of cancer patients and individuals from the general population. Soc. Sci. Med..

[16] García-Llana H., Remor E., Selgas R. (2013). Adherence to treatment, emotional state and quality of life in patients with end-stage renal disease undergoing dialysis. Psicothema.

[17] Webster A.C., Nagler E.V., Morton R.L., Masson P. (2017). Chronic Kidney Disease. Lancet.

[18] Mills K.T., Xu Y., Zhang W., Bundy J.D., Chen C.-S., Kelly T.N., Chen J., He J. (2015). A systematic analysis of worldwide population-based data on the global burden of chronic kidney disease in 2010. Kidney Int..

[19] Tao S., Tao S., Cheng Y., Liu J., Ma L., Fu P. (2019). Effects of probiotic supplements on the progression of chronic kidney disease: A meta-analysis. Nephrology.

[20] Coronel F., Montenegro J., Selgas R., Celadilla O., Tejuca M. (2005). Manual práctico de Diálisis Peritoneal.

[21] Boateng E.A., East L. (2011). The Impact Of Dialysis Modality On Quality Of Life: A Systematic Review. J. Ren. Care.

[22] A Brown E., Johansson L., Farrington K., Gallagher H., Sensky T., Gordon F., Da Silva-Gane M., Beckett N., Hickson M. (2010). Broadening Options for Long-term Dialysis in the Elderly (BOLDE): Differences in quality of life on peritoneal dialysis compared to haemodialysis for older patients. Nephrol. Dial. Transplant..

[23] Dąbrowska-Bender M., Dykowska G., Żuk W., Milewska M., Staniszewska A. (2018). The impact on quality of life of dialysis patients with renal insufficiency. Patient Prefer. Adherence.

[24] Bargman J.M., François K. (2014). Evaluating the benefits of home-based peritoneal dialysis. Int. J. Nephrol. Renov. Dis..

[25] Griva K., Goh C.S., Kang W.C.A., Yu Z.L., Chan M.C., Wu S.Y., Krishnasamy T., Foo M. (2015). Quality of life and emotional distress in patients and burden in caregivers: A comparison between assisted peritoneal dialysis and self-care peritoneal dialysis. Qual. Life Res..

[26] Chang Y.-T., Hwang J.-S., Hung S.-Y., Tsai M.-S., Wu J.-L., Sung J.-M., Wang J.-D. (2016). Cost-effectiveness of hemodialysis and peritoneal dialysis: A national cohort study with 14 years follow-up and matched for comorbidities and propensity score. Sci. Rep..

[27] Mauro J.C.J., Tobalina J.A.M., González J.C.S. (2012). Employment in the patient with chronic kidney disease related to renal replacement therapy. Nefrologia.

[28] Ledebo I., Ronco C. (2008). The best dialysis therapy? Results from an international survey among nephrology professionals. NDT Plus.

[29] Muehrer R.J., Schatell D., Witten B., Gangnon R., Becker B.N., Hofmann R.M. (2011). Factors affecting employment at initiation of dialysis. Clin. J. Am. Soc. Nephrol..

[30] Manera K.E., Johnson D.W., Craig J.C., Shen J.I., Ruiz L., Wang A.Y., Yip T., Fung S., Tong M., Lee A. (2019). Patient and caregiver priorities for outcomes in peritoneal dialysis: Multinational nominal group technique study. Clin. J. Am. Soc. Nephrol..

[31] Curtin R.B., Johnson H.K., Schatell D. (2005). The peritoneal dialysis experience: Insights from long-term patients. Nephrol. Nurs. J. J. Am. Nephrol. Nurses’ Assoc..

[32] Tong A., Lesmana B., Johnson D.W., Wong G., Campbell D., Craig J.C. (2013). The Perspectives of Adults Living With Peritoneal Dialysis: Thematic Synthesis of Qualitative Studies. Am. J. Kidney Dis..

[33] Howell M., Walker R.C., Howard K. (2019). Cost Effectiveness of Dialysis Modalities: A Systematic Review of Economic Evaluations. Appl. Health Econ. Health Policy.

[34] Pike E., Hamidi V., Ringerike T., Wisloff T., Klemp M. (2016). More Use of Peritoneal Dialysis Gives Significant Savings: A Systematic Review and Health Economic Decision Model. J. Clin. Med. Res..

[35] Vargas F. (2015). Documento arco sobre Enfermedad Renal Crónica (ERC) dentro de la estrategia de abordaje a la cronicidad en el SNS.

[36] Peipert J.D., Bentler P.M., Klicko K., Hays R.D. (2018). Psychometric Properties of the Kidney Disease Quality of Life 36-Item Short-Form Survey (KDQOL-36) in the United States. Am. J. Kidney Dis..

[37] Peipert J.D., Nair D., Klicko K., Schatell D.R., Hays R.D. (2019). Kidney Disease Quality of Life 36-Item Short Form Survey (KDQOL-36) Normative Values for the United States Dialysis Population and New Single Summary Score. J. Am. Soc. Nephrol..

[38] D’Onofrio G., Simeoni M., Rizza P., Caroleo M., Capria M., Mazzitello G., Sacco T., Mazzuca E., Panzino M.T., Cerantonio A. (2016). Quality of life, clinical outcome, personality and coping in chronic hemodialysis patients. Ren. Fail..

[39] Zazzeroni L., Pasquinelli G., Nanni E., Cremonini V., Rubbi I. (2017). Comparison of Quality of Life in Patients Undergoing Hemodialysis and Peritoneal Dialysis: A Systematic Review and Meta-Analysis. Kidney Blood Press. Res..

[40] De Brito D.C.S., Machado E.L., Reis I.A., Moreira D.P., Nébias T.H.M., Cherchiglia M.L. (2019). Modality transition on renal replacement therapy and quality of life of patients: A 10-year follow-up cohort study. Qual. Life Res..

[41] A Pagels A., Söderquist B.K., Heiwe S. (2015). Differences In Illness Representations In Patients With Chronic Kidney Disease. J. Ren. Care.

[42] Lin X., Shang Y., Teng S., Liu H., Han L. (2015). Relationship between Perceived Social Support and Quality of Life among Kidney Transplant Recipients. GSTF J. Nurs. Health Care.

[43] Da Silva S.M., Braido N.F., Ottaviani A.C., Gesualdo G.D., Zazzetta M.S., Orlandi F.D.S. (2016). Social support of adults and elderly with chronic kidney disease on dialysis. Rev. Latino-Am. Enferm..

[44] Eckhardt A.L., Devon H.A. (2017). The MIXED framework: A novel approach to evaluating mixed-methods rigor. Nurs. Inq..

[45] Holloway I., Galvin K. (2016). Qualitative Research in Nursing and Healthcare.

[46] Hays R., Kallich J., Mapes D., Coons S., Carter B. (1994). Development of the kidney disease quality of life (KDQOL TM) instrument. Qual. Life Res..

[47] Álvarez-Ude F., Galán P., Vicente E., Álamo C., Fernández-Reyes M., Badía X. (1997). Adaptación transcultural y validación preliminar de la versión española del Kidney Disease Questionnaire (Cuestionario de la enfermedad renal). Nefrología.

[48] Sherbourne C.D., Stewart A.L. (1991). The MOS social support survey. Soc. Sci. Med..

[49] Revilla L., Luna J., Bailón E., Medina I. (2005). Validación del cuestionario MOS de apoyo social en Atención Primaria. Med. Fam..

[50] Bardin L. (1977). Análise de conteúdo. Lisb. Edições.

[51] Park J.Y., Yoo K.D., Kim Y.C., Kim D.K., Joo K.W., Kang S.-W., Yang C.W., Kim N.-H., Lim C.-S., Kim Y.S. (2017). Early dialysis initiation does not improve clinical outcomes in elderly end-stage renal disease patients: A multicenter prospective cohort study. PLoS ONE.

[52] Fernández G.M., Cerrato A.O., Iniesta L.D.L.V., Galera E.O., Roldán C.G., Martínez J.P. (2016). Comparación entre bioimpedancia espectroscópica y fórmula de Watson para medición de volumen corporal en pacientes en diálisis peritoneal. Nefrología.

[53] Remón C., Quirós P., Portolés J., Gómez C., Carrasco A., Borràs M., Rodríguez C. (2014). Resultados del trabajo cooperativo de los registros españoles de diálisis peritoneal: Análisis de 12 años de seguimiento. Nefrología.

[54] López A.G., Nava Rebollo Á., Martín B.A., Herrera-Gómez F., Zapatero H.S., Martín J.D., Fernández F.C., Martínez Á.C., Muga C.E., Molina H.D. (2016). Grado de adherencia y conocimiento previo a la conciliación terapéutica en pacientes en diálisis peritoneal. Nefrología.

[55] Law M.C., Chow K.M., Fung J.S.F., Szeto C.-C., Li P.K.-T. (2016). Employment status in peritoneal-dialysis patients. Hong Kong J. Nephrol..

[56] Heras M., Guerrero M.T., Fernández-Reyes M.J., Muñoz A. (2017). Enfermedad renal «oculta» en ancianos: ¿deja de ocultarse a los 10 años de seguimiento?. Nefrología.

[57] Perl J., McArthur E., Bell C., Garg A.X., Bargman J.M., Chan C.T., Harel S., Li L., Jain A.K., Nash D.M. (2017). Dialysis Modality and Readmission Following Hospital Discharge: A Population-Based Cohort Study. Am. J. Kidney Dis..

[58] Almutary H., Bonner A., Douglas C. (2016). Which patients with chronic kidney disease have the greatest symptom burden? A comparative study of advanced ckd stage and dialysis modality. J. Ren. Care.

[59] Lim H.A., Yu Z., Kang A.W., Foo M.W.Y., Griva K. (2015). The Course of Quality of Life in Patients on Peritoneal Dialysis: A 12-month Prospective Observational Cohort Study. Int. J. Behav. Med..

[60] Gonçalves F.A., Dalosso I.F., Borba J.M.C., Bucaneve J., Valério N.M.P., Okamoto C.T., Bucharles S.G.E. (2015). Quality of life in chronic renal patients on hemodialysis or peritoneal dialysis: A comparative study in a referral service of Curitiba - PR. Braz. J. Nephrol..

[61] Griva K., Kang A.W., Yu Z.L., Lee V.Y.W., Zarogianis S., Chan M.C., Foo M. (2016). Predicting technique and patient survival over 12 months in peritoneal dialysis: The role of anxiety and depression. Int. Urol. Nephrol..

[62] Tannor E.K., Archer E., Kapembwa K., Van Schalkwyk S.C., Davids M.R. (2017). Quality of life in patients on chronic dialysis in South Africa: A comparative mixed methods study. BMC Nephrol..

[63] Zouari L., Omri S., Turki S., Mâalej M., Charfi N., Ben Thabet J., Mahfoudh H., Hachicha J., Maâlej M. (2016). Quality of life in chronic hemodialysis patients:about 71 cases. La Tunis. Med..

[64] Ghiasi B., Sarokhani D., Dehkordi A.H., Sayehmiri K., Heidari M.H. (2018). Quality of Life of patients with chronic kidney disease in Iran: Systematic Review and Meta-analysis. Indian J. Palliat. Care.

[65] Kurowski A., Czyzewski L., Sańko-Resmer J., Wyzgał J. (2014). Assessment of Health-Related Quality of Life of Patients after Kidney Transplantation in Comparison with Hemodialysis and Peritoneal Dialysis. Ann. Transplant..

[66] Park J.I., Kim M., Kim H., An J.N., Lee J., Yang S.H., Cho J.-H., Kim Y.-L., Park K.-S., Oh Y.K. (2015). Not Early Referral but Planned Dialysis Improves Quality of Life and Depression in Newly Diagnosed End Stage Renal Disease Patients: A Prospective Cohort Study in Korea. PLoS ONE.

[67] Oliveira A.P.B., Schmidt D.B., Amatneeks T.M., Dos Santos J.C., Cavallet L.H.R., Michel R.B. (2016). Quality of life in hemodialysis patients and the relationship with mortality, hospitalizations and poor treatment adherence. Braz. J. Nephrol..

[68] Vilagut G., Valderas J.M., Ferrer M., Garin O., López-García E., Alonso J. (2008). Interpretación de los cuestionarios de salud SF-36 y SF-12 en España: Componentes físico y mental. Med. Clínica.

[69] Doosti-Irani A., Nedjat S., Nedjat S., Cheraghi P., Cheraghi Z. (2018). Quality of life in Iranian elderly population using the SF-36 questionnaire: Systematic review and meta-analysis. East. Mediterr. Health J..

[70] Chen Y.-C., Chang L.-C., Liu C.-Y., Ho Y.-F., Weng S.-C., Tsai T.-I. (2018). The Roles of Social Support and Health Literacy in Self-Management Among Patients With Chronic Kidney Disease. J. Nurs. Sch..

[71] Li J., Wu X., Lin J., Zou D., Yang X., Cheng S., Guo Q. (2016). Type D personality, illness perception, social support and quality of life in continuous ambulatory peritoneal dialysis patients. Psychol. Health Med..

[72] Wang Q., Yang Z.-K., Sun X.-M., Du Y., Song Y.-F., Ren Y.-P., Dong J. (2017). Association of Social Support and Family Environment with Cognitive Function in Peritoneal Dialysis Patients. Perit. Dial. Int..

[73] Van Hoang L., Green T., Bonner A. (2018). Informal caregivers’ experiences of caring for people receiving dialysis: A mixed-methods systematic review. J. Ren. Care.

