# Supplementary material for: Quality of Life and Social Support of People on Peritoneal Dialysis: Mixed Methods Research
Source: Int J Environ Res Public Health. 2020 Jun 14;17(12):4240. doi: 10.3390/ijerph17124240 (PMC7345330; doi:10.3390/ijerph17124240)
Supplement: Supplementary file 1 [file ijerph-17-04240-s001.pdf]

Table S1: Relationship between the physical limitations dimension of the KDQOL-SF™ instrument and the social support dimensions of the MOS survey

**KDQOL-SF™(physical limitations dimensions)**	**Emotional and informational support**	***p***	**Instrumental support and support material**	***p***	**Positive social interaction**	***p***	**Affective support**	***p***
Had to reduce the time dedicated to work or his or her daily activities								
Yes (n:19)<br>*No* (n:36)	4.03 (0.93)<br>4.19 (0.91)	0.337	4.26 (1.09)<br>4.43 (1.10)	0.088	3.71 (1.01)<br>3.75 (1.19)	0.662	4.22 (0.92)<br>4.24 (1.14)	0.508
Did less than he or she would have liked								
Yes (n:29)<br>*No* (n:26)	3.87 (0.93)<br>4.43 (0.80)	0.011	4.03 (1.26)<br>4.75 (0.73)	0.001	3.44 (1.14)<br>4.06 (1.02)	0.033	3.93 (1.03)<br>4.57 (1.02)	0.001
Had to stop doing some tasks in his or her work or daily activities								
Yes (n:20)<br>*No (n:35)*	3.79 (0.92)<br>4.33 (0.85)	0.013	3.77 (1.37)<br>4.71 (0.72)	<0.001	3.47 (0.98)<br>3.89 (1.11)	0.084	3.78 (1.09)<br>4.49 (0.97)	0.003
Had difficulty in doing his or her work or daily activities (e.g. it took more effort than normal)								
Yes (n:25)<br>*No (n:30)*	3.78 (1)<br>4.43 (0.72)	0.010	4.07 (1.25)<br>4.62 (0.88)	0.008	3.25 (1.17)<br>4.15 (0.91)	0.004	3.84 (1.04)<br>4.56 (0.99)	0.001

*Mann–Whitney U test *The quantitative variables are described with the mean and with the standard deviation in parentheses*

Table S2: Relationship between the emotional limitation dimensions of the KDQOL-SF™ instrument and the social support dimensions of the MOS survey

**KDQOL-SF™(emotional limitations dimension)**	**Emotional and informational support**	***p***	**Instrumental support and support material**	***p***	**Positive social interaction**	***p***	**Affective support**	***p***
Had to reduce the time dedicated to work or daily activities due to an emotional problem								
Yes (n:13)<br>No (n:42)	3.58 (0.83)<br>4.30 (0.87)	0.005	3.59 (1.50)<br>4.61 (0.81)	0.005	3.28 (0.97)<br>3.88 (1.13)	0.053	3.35 (1.10)<br>4.50 (0.91)	0.000
Did less than he or she would have wanted due to an emotional problem								
Yes (n:12)<br>No (n:43)	3.77 (0.83)<br>4.24 (0.91)	0.043	3.68 (1.53)<br>4.56 (0.86)	0.018	3.37 (0.94)<br>3.84 (1.15)	0.116	3.58 (1.20)<br>4.41 (0.96)	0.013
Had to stop doing some tasks at work or in his or her daily activities due to an emotional problem								
Yes (n:13)<br>*No (n:42)*	4.10 (0.83)<br>4.14 (0.94)	0.637	3.80 (1.55)<br>4.54 (0.85)	0.216	3.51 (1.06)<br>3.80 (1.14)	0.318	4.05 (1.10)<br>4.29 (1.06)	0.308

*Mann–Whitney U test *The quantitative variables are described with the mean and with the standard deviation in parentheses*

Table S3: Correlation between the physical and mental component of the SF-36, emotional support, instrumental support, affective support, cognitive function, sexual function, problems and symptoms, discomforting effects of the kidney disease, the burden of the kidney disease and social support

	**1**	**2**	**3**	**4**	**5**	**6**	**7**	**8**	**9**	**10**	**11**
**1.Physical comp.SF-36**	1	0.6	0.284*	0.127	0.337*	-0.397**	0.107	0.437**	0.344*	0.288*	0.233
**2.Mental comp. SF-36**		1	0.388**	0.24	0.306*	-0.586**	0.25	0.364**	0.611**	0.649**	0.407**
**3.Emocional support**			1	0.367**	0.759**	-0.357**	0.26	0.155	0.389**	0.290*	0.599**
**4. Instrumental support**				1	0.479**	-0.262	0.276*	0.241	0.212	0.051	0.442**
**5.Affective support**					1	-0.417**	0.203	0.249	0.298*	0.268*	0.646**
**6. Cognitive function**						1	-0.332*	-0.595**	-0.579**	-0.463**	-0.386**
**7.Sexual function**							1	0.151	0.628**	0.194	0.277*
**8.Problems and symptoms**								1	0.477**	0.305*	0.26
**9.Discomforting effects**									1	0.602**	0.380**
**10.Burden of kidney disease**										1	0.235
**11.Social support**											1

*Spearman's Rho test * Statistical significance p<0.05; ** Statistical significance p<0.001*
